# Anticipating the impact of COVID19 and comorbidities on the South African healthcare system by agent-based simulations

**DOI:** 10.1038/s41598-021-86580-w

**Published:** 2021-04-12

**Authors:** Jan Christian Schlüter, Leif Sörensen, Andreas Bossert, Moritz Kersting, Wieland Staab, Benjamin Wacker

**Affiliations:** 1 Next Generation Mobility Group, Department of Dynamics of Complex Fluids, Max Planck Institute for Dynamics and Self-Organization, Am Fassberg 17, 37077 Göttingen, Germany; 2 Institute for the Dynamics of Complex Systems, Faculty of Physics, Georg August University of Göttingen, Friedrich-Hund-Platz 1, 37077 Göttingen, Germany; 3 Institute for Medical Epidemiology, Biometrics and Informatics, Interdisciplinary Center for Health Sciences, Martin Luther University Halle Wittenberg, Magdeburger Straße 8, 06112 Halle, Germany; 4 Thinktank of Aeronautics, Aerodynamics and Aerospace Technology, Marschblick 5, 25866 Mildstedt, Germany; 5 Center of Methods in Social Sciences, Department of Social Sciences, Georg-August-University of Göttingen, Goßlerstraße 19, 37073 Göttingen, Germany; 6 Chair of Regional Management and Business Promotion, Faculty of Resource Management, HAWK University for Applied Sciences and Art, Büsgenweg 1a, 37077 Göttingen, Germany; 7 University Medical Center Wilhelmshaven, Wilhelmshaven, Germany; 8 Diagnostic and Interventional Radiology, University Medical Center Goettingen, Göttingen, Germany; 9 Department of Engineering and Natural Sciences, University of Applied Sciences Merseburg, Eberhard-Leibnitz-Str. 2, 06217 Merseburg, Germany

**Keywords:** Diseases, Mathematics and computing

## Abstract

Tuberculosis (TB) is the 10th leading cause of death worldwide, and since 2007 it has been the main cause of death from a single infectious agent, ranking above HIV/AIDS. The current COVID-19 is a pandemic which caused many deaths around the world. The danger is not only a coinfection as observed for TB and HIV for a long time, but that both TB and SARS-CoV-2 affect the respiratory organs and thus potentiate their effect or accelerate the critical course. A key public health priority during the emergence of a novel pathogen is the estimation of the clinical need to assure adequate medical treatment. This requires a correct adjustment to the critical case detection rate and the prediction of possible scenarios based on known patterns. The African continent faces constraining preconditions in regard to healthcare capacities and social welfare which may hinder required countermeasures. However, given the high TB prevalence rates, COVID-19 may show a particular severe course in respective African countries, e.g. South Africa. Using WHO’s TB and public infrastructure data, we conservatively estimate that the symptomatic critical case rate, which affects the healthcare system, is between 8 and 12% due to the interaction of COVID-19 and TB, for a TB population of 0.52% in South Africa. This TB prevalence leads to a significant increase in the peak load of critical cases of COVID-19 patients and potentially exceeds current healthcare capacities.

## Introduction

After initial infections with an unknown respiratory disease in late December 2019, the new SARS-CoV-2 was identified on 9 January 2020 as the cause for COVID-19^1^. With the epicentre in Wuhan, China, COVID-19 spread at a rapid pace around the globe. In consequence, it was declared a pandemic by the WHO on 11 March 2020^2^. Governments and international organisations elaborate on appropriate countermeasures to contain the COVID-19 pandemic. For some countries the current number of cases is already bringing their health systems to the brink of collapse^3^. To provide relief to the medical infrastructure and to slow down the growth of infection rates, non-pharmaceutical countermeasures have been imposed through more or less restrictive actions worldwide. Apart from severe human costs, these measures also come at high economic costs^4^ and may pose a particular burden on developing countries, e.g. on the African continent^5,6^. Under these circumstances patients suffering from comorbidities may not be assured required treatment once African countries face increasing COVID-19 infection numbers in the next months^7,8^. At the time of writing, infection numbers in WHO African Region remain comparatively low with 47,953 infections by 12 May 2020 which is a 42% increase over the week before according to the WHO^9^. Deaths stood at 1488 resulting in a case fatality ratio of 3.1%. However, there has been indication of underestimating the infection rate and actual numbers^10^. The actual number of infected people on the African continent is likely to be higher than currently stated^11^. A potential reason is the low number in tests per day in several countries, e.g. a little more than 5000 daily tests over a target volume of 10,000–15,000 in South Africa. Thus, these countries are likely to face a rapid increase in infections over the coming weeks and require effective countermeasures.

South Africa has reacted quickly to the spread of the virus with a strict nationwide lockdown to keep the burden on the medical infrastructure low. The strict lockdown showed a decisive effect on infection rates and the South African government has been credited for its effective countermeasures. The reason for its momentarily good performance may be related to experiences from tackling contagious diseases in the past, e.g. through active case finding in vulnerable communities by more than 28,000 community workers. However, from a medical point of view, South Africa might still face a high risk for increasing infection and fatality rates as it has the fourth highest number of tuberculosis (TB) cases per capita^12^, a high HIV prevalence and a high rate of people infected with both HIV and TB. South Africa is found on three WHO lists for TB, TB/HIV and MDR-TB high burden countries accounting for 3% of total cases worldwide^12^. In 2018, the total TB incidents for South Africa stood at 520 per 100.000 inhabitants. Consequently, treatment of COVID-19 may come at the cost of reduced but required treatment for other infectious diseases^7^. Moreover, lack of access to medical services may further aggravate the situation for the most vulnerable population segments in the coming winter period^13^.

On 5 March 2020 the first confirmed case of COVID-19 was reported in South Africa^14^. The President declared state of disaster which implied restrictions on public life on March 15, up to which point South Africa had 61 confirmed COVID-19 cases^15^. Major restrictions on public life followed 3 days later with the closing of schools, parliament and most universities. A nationwide lockdown was imposed on March 27, initially for a duration of 21 days, which was prolonged on April 9 until the end of April 2020^16^. Essential workers are exempt from these measures to support the tackling of the COVID-19. Borders are closed and the South African government is evacuating South Africans from foreign countries. South Africa has conceived broad attention for its strict countermeasures which prohibit most outdoor activities except everyday necessities. Despite criticism on the strict enforcement of the measures^17^, rigid lockdowns have shown positive results regarding the mitigation of COVID-19 spread elsewhere^18^.

The viral pneumonia, SARS-CoV-2, that spread from Wuhan, China, to many parts of the world in 2019, leads to mainly mild symptoms in the general population but may result in severe outcomes for people with underlying diseases such as HIV/AIDS and/or TB^19^. Worldwide about 900,000–1,100,000 people die due to HIV/AIDS infection and an estimated 1.8 million newly HIV/AIDS infections are suspected every year. Of these, about 70 % are based in the African region^20,21^. Tuberculosis is the leading cause of death from a single infectious agent worldwide, ranking above HIV/AIDS. More than 25 % of these tuberculosis associated deaths occur in the African Region^20,22^. Non-treated HIV/AIDS infection causes a progressive dysfunction of surviving lymphocytes and additionally will result in a decline of CD4+ T lymphocytes. Furthermore, for people who suffer from a latent infection with *Mycobacterium tuberculosis*, an additional HIV infection is the most severe known risk factor for progression to active TB. For people tested positive for HIV and additionally infected with Mycobacterium tuberculosis, the annual risk of developing active disease is up to 15 %, with a lifetime risk of 50 % or more^22,23^.

To our knowledge, little research exists on the potential joint effects of the new COVID-19 pandemic and TB prevalence on severe and critical infection rates^24^. As a study from China indicates there are potential effects of TB on an increase of susceptibility for COVID-19^19^. By including TB penetration in SIR models, we have conducted an agent-based simulation for South Africa to simulate the spread and the associated burden of COVID-19 in combination with TB on the healthcare system. The estimation of potential case numbers^25^ can be relevant to prepare medical infrastructure for worst potential outcomes.

We assume, that these underlying diseases will result in a far more severe reaction on SARS-COV-2 infection, especially in the African region due to a wide spread general dissemination of HIV/AIDS and Mycobacterium tuberculosis related tuberculosis. We propose that risk groups, e.g. TB patients, increase the relevance of adequate measures against the COVID-19 pandemic. While HIV/Aids is assumed to show no causal relationship with the current new SARS-CoV-2 by the time of writing^26–28^, TB implies a potentially high vulnerability of infected people. While the importance of continuity in TB treatment has been emphasised^8^, there has been doubt about whether this states a realistic aim^29^. The main concern in high TB-prevalence countries are discontinued treatments and fewer medical tests which may result in a higher TB incidence over the next years^30^. A similar approach indicates that TB-related deaths might increase over the next 5 years in low- and middle-income countries due to longer periods without proper treatment and higher infection figures^31^. COVID-19 might, therefore, increase the number of deaths by up to 20 % in the worst-case scenario in their numeric simulations arguing strongly in favour of continued treatments. For China, current research indicates that a TB response mechanism should be implemented to cope with delayed TB cases and treatments during the COVID-19 pandemic^32^.These studies as well as this paper point towards potential negative outcomes of COVID-19 in countries with a high TB-prevalence the future impact may prove an even more severe outcome.

In contrast, several contributions point towards potential positive effects of COVID-19 on TB treatment in South Africa as the pandemic reveals inefficiencies in the status-quo of TB treatment which could be changed over the next years^33,34^.

Under the premise that infections are likely to increase over the coming weeks and months and that TB and COVID-19 may lead to a surge of critical cases, we assess the potential results of simulations under consideration of TB in a South African region.

## Method

We show results from an agent-based epidemiological modified transport simulation where we included the proportion of TB infected in Nelson Mandela Bay Municipality (NMBM) in South Africa according to socio-demographic data. We focus the analysis on potential effects on the South African healthcare system under consideration of the TB risk group. TB patients were declared *high-risk* patients in our simulation.

In general, such a simulation will allow:

- faster detection of transmission zones/areas and pathways.
- Identification of regions with high potential for dissemination.
- Prediction of future regional spread and evaluation of effectiveness of the various countermeasures on number of symptomatic persons.

We hypothesise that TB prevalence combined with the new SARS-CoV-2 results in an increased number of severely sick or even critical patients, i.e. patients that require (intensive) medical treatment. Under these circumstances, available intensive care (ICU) units and the required supportive infrastructure such as staff and equipment may be insufficient resulting in an increased mortality rate^35^. Consequently, our hypothesis implies that TB prevalence aggravates the influence of COVID-19 on healthcare capacities in NMBM. Thus, these results could be a valuable tool for decision makers to assess adequate regional countermeasures and general pandemic preparedness.

MATSim is a multiagent transportation simulation framework, that models movements and activities of synthetic agents within a road network^36^. A synthetic population consists of multiple independent agents that move in a network of e.g. roads, places and buildings to complete their individually designated tasks such as working, shopping, staying at home or performing leisure activities during a day. In order to obtain a realistic simulation of a region, geo- and census- time-use or similar data are usually processed. For the NMBM, both a network and a synthetic population representing 10 % of the inhabitants processed from demographic data are taken from previous publications^37^. Since the agents are generated from census data and instructed to behave rationally, their movements and especially their contacts with other agents represent a realistic approximation of human daily routines.

Depending on the simulation, each agent can choose from a variety of means of transport (e.g. walking, car, bus, taxis) to reach his destinations. The agents are motivated by a utility function to make efficient choices, since some circumstances such as paying high fares, being late at work or spending much time in a traffic jam are punished and others, such as performing leisure activities are rewarded.

As recent contributions^38–40^ suggest, such agent-based traffic simulations might prove as a useful tool for other purposes such as epidemic simulations. Assuming a sufficient data basis, e.g. geographical or demographic conditions of a region can be related to the expected course of an epidemic. The movements of the agents within the network are trackable and thus the duration and intensity of their contacts to other agents in buildings, vehicles or other places can be measured or estimated. These daily trajectories allow to compute infection chains based on stochastic infection events.

Episim was initially set up for the city of Berlin^38^ and is an extension for MATSim. It is based on two major components as it simulates *infection events* on a parametred stochastic function and calculates the *progress* of an infected agent’s disease based on an extended SIR model. At the beginning of the simulations, several agents become initially infected to start the epidemics. Further infections occur based on a probabilistic function of determined infection parameters. Agent *n* is infected at time *t* with probability

1 $$P_{n,t} = 1 - {\text{exp}}[ - \theta \mathop \sum \limits_{m} q_{m,t} i_{nm,t} \tau_{nm,t} ],$$

where *q* denotes the shedding rate (infectivity-parameter for the virus), *i* a contact intensity parameter and $\tau$ the interaction duration of agents. *i* is assumed to exponentially decline by distance. The infection parameters and the model calibration correspond to previous publications^39,40^ in order to fit the infection growth rates during the first weeks.

In the original framework, the course of disease is equal for all infected with 4.5% randomly selected of the infected becoming seriously sick. Of those, 25% become critical and require an ICU. As COVID-19 is known for numerous very mild cases 20% of the infected are assumed to notice their infection and go into self-quarantine. For the case of South Africa, the present study assumes 0.52% of the population to be infected with TB and thus belong to a group with an increased risk for severe illness during a COVID-19 infection. For the sake of simplicity, the present study does not differentiate between different stages or forms of the disease but assumes a uniform susceptibility, infectivity and probability of a severe course for all tuberculosis patients. Furthermore, demographic characteristics such as age or economic status are not explicitly considered in the epidemic simulation, but are based on generalised parameters. However, they are implicitly considered in the transport simulation and thus influence the social contacts of the agents and possible infection events. These simplifications do not compromise the results, as the infection process is simulated independently of the course of the disease and the large number of agents levels out fluctuations. Once infected with COVID-19, a share of approximately 21.15% of this risk group is assumed to become critical patients. The values are based on the TB incidence and TB mortality as estimated by the WHO^12^. Assuming that within the TB group only cases with fatal outcome require an ICU is a conservative approach and might underestimate real ICU demand. Similarly, current estimates indicate a gap in available and required healthcare units (especially ICUs) for the next months in the U.S.^41^.

To evaluate the possible influence of comorbidities on the critical case numbers in the course of the corona epidemic, several scenarios with varying rates were simulated for each co-occurring condition and the proportion of seriously ill infected persons with comorbidities. Change rates of critical cases with respect to alternating parameters are computed as follows:

2 $$\overline{C}_{d} = \frac{1}{10}{*}\mathop \sum \limits_{r = 1}^{10} C_{r,d}$$

3 $$\overline{I}_{d} = \frac{1}{10}{*}\mathop \sum \limits_{r = 1}^{10} I_{r,d}$$

4 $$\widetilde{{\overline{C}}} = \frac{1}{D}{*}\mathop \sum \limits_{d = 1}^{D} \overline{C}_{d} v ; D \in \left( {1;365} \right)$$

5 $$\widetilde{{\overline{I}}} = \frac{1}{D}{*}\mathop \sum \limits_{d = 1}^{D} \overline{I}_{d} v ; D \in \left( {1;365} \right)$$

6 $$e_{i,j} = \frac{{\widetilde{{\overline{C}}}_{j,i} }}{{\widetilde{{\overline{I}}}_{j,i} }}{* }\frac{{\widetilde{{\overline{I}}}_{j,1} }}{{\widetilde{{\overline{C}}}_{j,1} }}{ };{ }\widetilde{{\overline{C}}}_{j,i} \subset \widetilde{{\overline{I}}}_{j,i}$$

where $C_{r,d}$ denotes the number of critical cases and $I_{r,d}$ the number of Infections in realisation r on day d. $\overline{C}_{d}$ and $\overline{I}_{d}$ represent the corresponding daily arithmetic means of ten realisations. Moreover, $\overline{C}_{d}$ and $\overline{I}_{d}$ are the averages of those means over time. D denotes either the last day of the epidemic or day 365, since the simulations are set up to terminate after one year.The elements *e *_*ji*_ in Table 2 are calculated based on equation 8, where *i* denotes 11 TB-rates ranging from 0 to 1% and *j* the share of critical cases among TB-patients in 6 steps from 0 to 25%.

For all analyses, three scenarios are conducted to assess the impact of different policy measures on the epidemic’s course. An initial lockdown of 25 days is either lifted (scenario A), maintained (scenario B) or tightened (scenario C). Episim allows to reduce the prevalence of any activity within the simulation. Thus, a reduction of e.g. public leisure activities, the use of public transport or education can be simulated. The remaining share of each activity in the different lockdown scenarios is depicted in Table 1.

**Table 1:** Simulation setups including the allowed share of an activity type of all activity types after a certain lockdown.

Scenario	Home (%)	Work (%)	Minibus (%)	Leisure taxis (%)	Primary education (%)^a^	Higher education (%)	Shop (%)	Dropby (%)	Other (%)
A (lift lockdown)	100	100	100	100	100	100	100	100	100
B (maintain lockdown)	100	15	50	0	0	0	30	0	15
C (tighten lockdown)	100	5	0	0	0	0	10	0	10

^a^Including kindergartens.

## Simulation results

**Figure 1:**
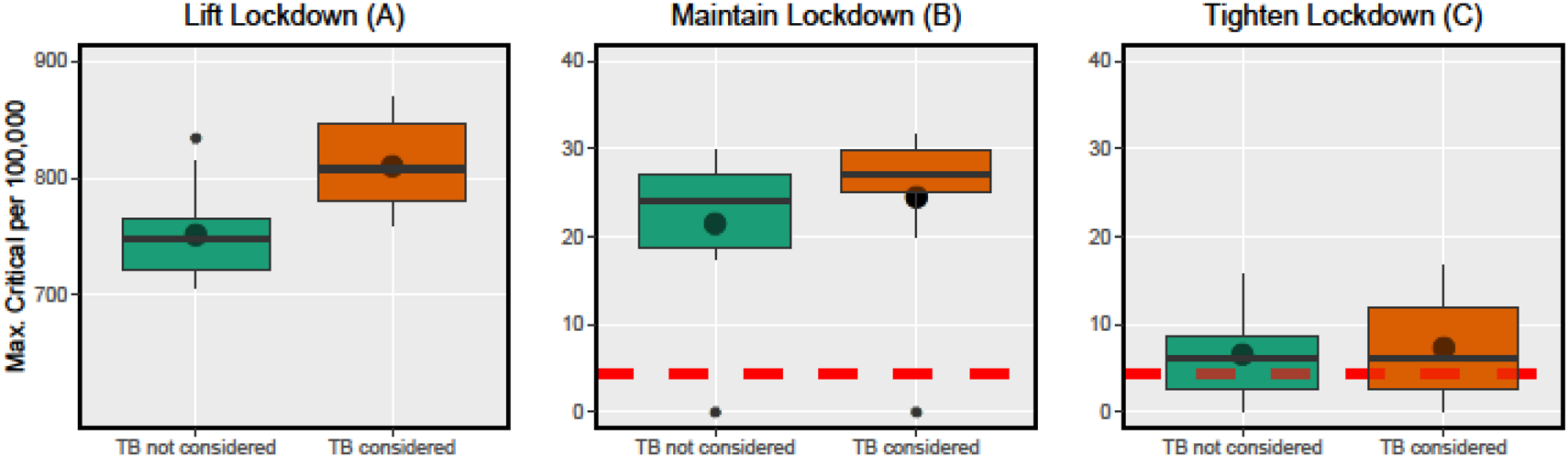
Policy impacts on peaks of critical cases (summary). Properties of the maximum number of critical cases throughout the epidemics for 130 days. The three policy scenarios are conducted and model lifting, maintaining and tightening the lockdown. Each scenario is simulated with and without consideration of TB as a risk factor to assess the impact. All policy scenarios start with 100 infected inhabitants and a default lockdown is applied until day 25. The dashed red lines represent the number of available ICUs. Each setup is based on ten simulations.

Figure 1 summarises our simulations of the critical COVID-19 cases in NMBM for different scenarios per 100,000 inhabitants and the impact of the TB subpopulation on them. TB increases the arithmetic mean of critical cases in each scenario (A: 8%, B: 14%, C: 11%). In scenario *A*, the number of critical cases increases from 748 in the median by 8% to 808 per 100.000 inhabitants and the interquartile range increases by about 52%. When the measures are adjusted to scenario *B*, the number of critical cases increases from 24 in the median by 13% to 27 per 100.000 inhabitants and the interquartile range decreases by 39%. With an increase of the restrictions in scenario *C*, the number of critical cases remains constant at 6 per 100.000 population in the median and the interquartile range increases by 59%. Overall, the 3rd quartiles of the boxes in TB are all at a higher level than without TB (A: 11%, B: 10%, C: 41%).

We further assessed the influence of different proportions of TB in the population and its epidemiological effects associated with COVID-19 in NMBM (see Table 2).

**Table 2:** Relative impacts of different disease-parameters on the mean of critical cases for the course of the disease.

**Share seriously sick (of TB, %)**						
TB-rate %	0	5	10	15	20	25
*A*						
0	1	1	1	1	1	1
0.1	1	1.03035	1.01633	1.00999	1.03993	1.02155
0.2	1	1.01242	1.00785	1.009	1.03939	1.04864
0.3	1	1.01004	1.03329	1.02252	1.05993	1.04588
0.4	1	1.0276	1.0293	1.042	1.06465	1.08001
0.5	1	1.0396	1.05828	1.07369	1.11453	1.10648
0.6	1	1.03496	1.05701	1.09342	1.11121	1.11831
0.7	1	1.01819	1.04924	1.06838	1.11287	1.15601
0.8	1	1.03813	1.07442	1.10708	1.15252	1.17524
0.9	1	1.01751	1.05305	1.09026	1.14126	1.17462
1.0	1	1.05061	1.09632	1.12924	1.17356	1.2281
*B*						
0	1	1	1	1	1	1
0.1	1	0.98975	1.00384	1.05478	1.02842	1.02733
0.2	1	0.97934	0.99778	1.01924	0.99915	1.0483
0.3	1	0.98555	1.02219	1.06228	1.06278	1.07647
0.4	1	1.00491	1.07643	1.07667	1.09182	1.07888
0.5	1	1.00524	1.02818	1.05523	1.11417	1.07978
0.6	1	1.01042	1.04876	1.09514	1.09227	1.11231
0.7	1	1.00563	1.03801	1.0542	1.09898	1.13496
0.8	1	1.03724	1.08618	1.11729	1.16716	1.19175
0.9	1	1.04281	1.04547	1.15125	1.1803	1.22339
1.0	1	1.0311	1.07369	1.15156	1.16997	1.23115
*C*						
0	1	1	1	1	1	1
0.1	1	1.01186	1.02033	1.02502	1.0199	1.03785
0.2	1	1.00332	1.01944	1.03463	1.03134	1.06007
0.3	1	1.01235	1.03673	1.04199	1.05067	1.0687
0.4	1	1.01935	1.02577	1.06918	1.05979	1.09914
0.5	1	1.01388	1.04056	1.06575	1.08742	1.10239
0.6	1	1.02555	1.04453	1.07094	1.09379	1.13729
0.7	1	1.03196	1.05095	1.08583	1.12673	1.13576
0.8	1	1.03391	1.05266	1.09875	1.13493	1.19383
0.9	1	1.04972	1.09667	1.12931	1.14672	1.18844
1.0	1	1.05472	1.09445	1.13935	1.17821	1.20686

The rows reflect on TB rates ranging from 0 to 1% of the population. The columns denote the share of COVID-19 infected TB patients that become seriously sick and cover a range from 0 to 25%. All values are calculated based on the first column as described in Eq. 8. The simulation ended either with the regeneration of the last infected agent or after 365 days. Simulations were carried out for the policy scenarios introduced above. The results show the impact of different TB rates and the proportion of seriously sick people among those infected with TB on the proportion of seriously sick people in the total population compared to a baseline scenario without TB.

**Figure 2:**
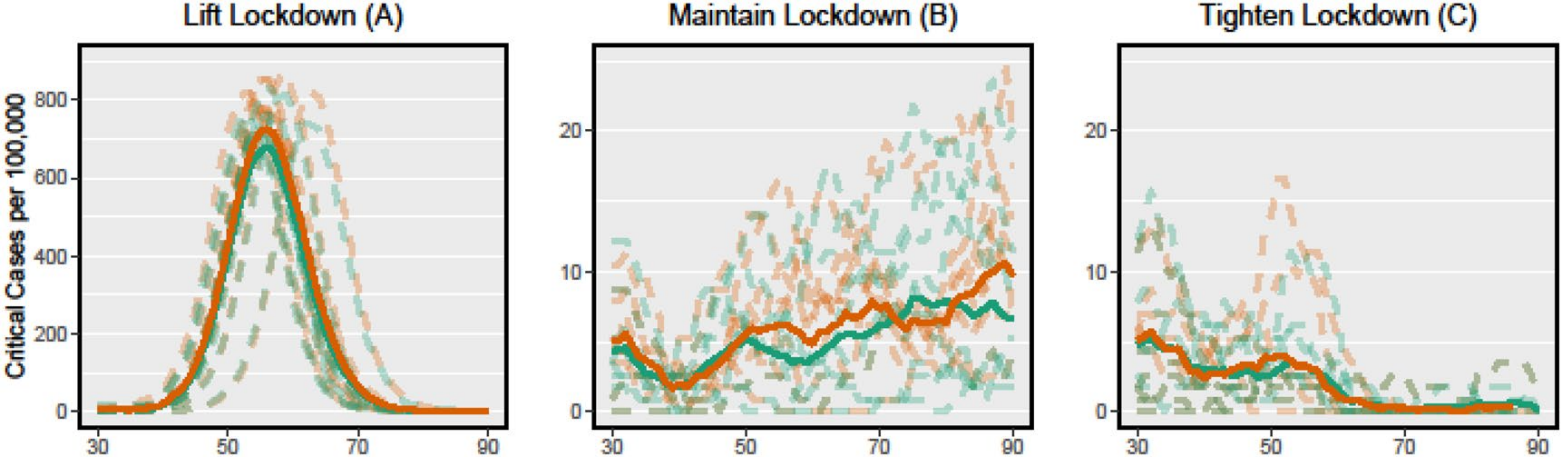
Policy impacts on the critical cases (time). Simulated course of COVID-19 in three policy scenarios corresponding to Fig. 1. The dashed green lines indicate the number of critical patients in scenarios without consideration of TB, the dashed red lines accordingly represent the critical cases under consideration of a TB risk group. For each scenario 10 simulations are computed, the solid lines represent their averages.

The increase in critical cases is linear to the proportion of the population with TB, assuming a constant mortality rate in South Africa. The different state interventions, as presented in Fig. 2, have a significant impact on the influence of the TB subpopulation: (A) It shows the regular increase of TB cases with a higher proportion of the subpopulation in the total population.

(B) The lockdown measures reduce the number of critical cases overall and reduce the impact of the TB subpopulation. (C) The strong lockdown measures greatly reduce the number of non-TB infected critical cases, resulting in a higher impact of the TB subpopulation on the critical cases.

**Figure 3:**
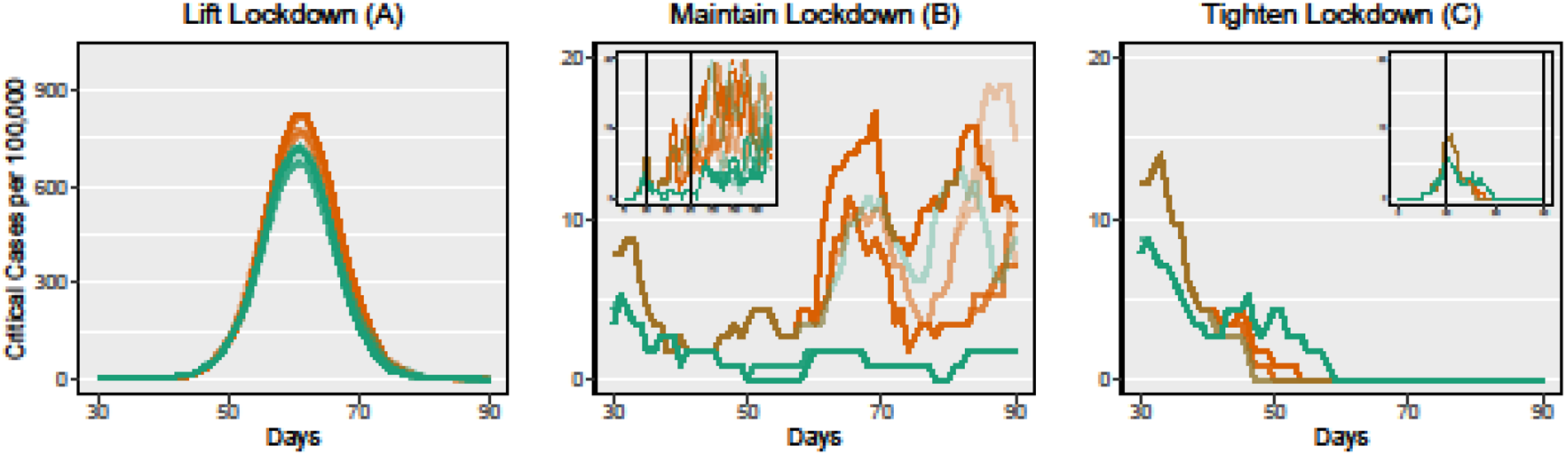
Varying TB rates. Each panel depicts 10 simulated courses of COVID-19 measured in critical cases per 100,000 inhabitants with a TB rate varying from 0% (green line) to 1% (red line). The small panels depict the course over a longer time horizon (200 days).

Figure 3 shows the influence of TB cases on the course of the epidemic. In the first phase of the epidemic, the mean course is similar to the mean course without TB. After about 25 days the TB course decouples and increases the number of critical cases significantly. At individual short periods of time, the number of critical cases without TB may be higher than with TB due to random fluctuations. However, the trend clearly decouples itself from the scenarios without TB and generally runs at a higher level.

## Discussion and conclusion

Based on the characteristics of TB as well as COVID-19, a disproportionate impact of TB related cases on COVID-19 critical cases is likely. To falsify this, we have adapted the simulations to regional conditions in NMBM, South Africa, including transport systems, mobility behaviour and plausible infections of agents with TB and COVID-19 in different scenarios. This influence may show impact on the regional healthcare system and the required ICU capacity for COVID-19 cases. The simulations suggest that current medical capacities of NMBM may be insufficient for coping with COVID-19 and TB patients simultaneously.

Unlike most analyses on COVID-19 and comorbidities, e.g. TB or HIV, our approach makes use of agent-based simulations rather than purely numeric or mathematical models^31,32^. These agent-based simulations take more area-specific characteristics into account than numerical simulations, thus, providing more specific results than generalised models. This approach further allows to evaluate certain COVID-19 countermeasures individually, e.g. by closing or opening specific local facilities.

Under the assumption that COVID-19 infections are likely among essential but poorly equipped healthcare staff, the necessity to quickly acquire more qualified medical staff is further increased^42^. Thus, a rapid treatment of TB patients before COVID-19 potentially spreads into vulnerable subpopulations is recommendable. This might be an obstacle given constrained public budgets for precautionary medical measures as well as looming increased COVID-19 related spending^43^.

The observed critical cases in NMBM may differ to other regions but give a first conservative estimate of the possible impact of TB on COVID-19 cases. Our results allow other scientists to compare the predictions and different proportions of the population with TB. The information generated and the future availability of data on TB and COVID-19 patients with a special focus on the African continent can be used to better understand these comorbidities and help policy makers in decision processes on counter- and precautionary measures.

At present the ratio of critically to moderately infected cases in South Africa ICU capacities are sufficient. However, the first cases arrived in South Africa through return travellers from Italy and potentially China^44^. The current COVID-19 infections may, thus, be prevalent in middle and higher income classes with a propensity to afford international travel. Moreover, the economic status may allow for adequate medical treatment and the required quarantine which lower income classes are less likely to be able to afford. Population in lower income classes may be neither able to refrain from working in order to assure a minimal income nor keep the required distance to their surroundings to reduce the likelihood to spread the COVID-19. In view of possibly lower test rates in townships, this harbours an unrecognised burden for the South African medical system, especially given the increased prevalence of comorbidities, such as HIV and TB.

As seen in countries like Italy, Spain and the U.S., COVID-19 may result in critical cases exceeding ICU capacity. A combination of high TB prevalence with the high number of COVID-19 infected people may exacerbate the situation resulting in insufficient treatment capacities. We have therefore adjusted our results to 100,000 inhabitants, so that comparable regions can correctly assess their ICU capacities as well as their TB subpopulation and mortality rates in order to take appropriate action.

Our model serves as a proxy for the impact of TB and COVID-19 on the healthcare system. Despite a comparatively high TB mortality rate, South Africa still has one of the best healthcare systems on the African continent.

In our approach we used TB fatality rates as estimates for the propensity of critical cases. Since the resulting fatality rate would have occurred regardless of COVID-19 our estimates can be considered to be rather conservative. Possible serious complications will only become visible in the coming weeks and we will adjust the parameters accordingly. It remains to be noted, however, that the regional healthcare system is already overburdened in any of our scenarios despite our conservative assumptions (see Fig. 1). Decision makers should therefore carefully consider further measures, especially with regard to TB infected patients.

While our findings indicate that TB is likely to increase the number of critical COVID-19 cases, in any case profound clinical data and research is necessary for falsification. Further clarification of the situation requires new data sources that are not yet available. Studies on the course of disease in COVID-19 and TB infected patients will reveal the actual consequences of comorbidity between TB and COVID-19 in the future.

With a subpopulation of 0.52% that is pre-infected with TB and thus is assumed to require an ICU, we can expect an increase in critical cases in this subpopulation of approximately 10% of the total population, even with strong community interventions (4.5% of the not TB-infected population become seriously sick and of that 25% critical).

In the context of this study it becomes clear that regions with TB prevalence should be given special measures to avoid the overburdening of the healthcare system, especially at the peak of the epidemic. The small number of people in these subpopulations should allow for measures to protect them and at the same time relieve the healthcare system as a whole. Potentially, the international community should assist these countries to lower the burden on the stressed healthcare systems if necessary^5^.

Several important reservations are worth mentioning for our simulations. First and most importantly, our simulations are estimates based on existing transport infrastructure, mobility data and assumptions on human behaviour. The model estimates are conservatively assumed and are inevitably affected by underperformance of TB cases and deaths under the influence of COVID-19. Given the current lack of definitive data elements on TB and COVID-19 cases, serial studies on comorbidity and mortality and behavioural changes due to governmental restrictions will be beneficial for future simulations.

For the prevalence estimates, we have used WHO data on TB for South Africa of 0.52% and a mean rate of 21.2% for the critical cases of infected TB cases, which is the mean fatality rate without exposure to COVID-19^12^. The upper and lower fatality rates for TB cases relative to the lower and upper TB cases in South Africa are 22.8% and 21.5% respectively. This means that we have adopted a conservative model for South Africa.

Regional socio-economic factors, such as a better developed healthcare system and regional treatment centres for TB-infected people, can lead to deviations from the national governmental data and thus result in diverging outcomes between countries and regions.

The applicability of these results to other TB affected countries is possible to a limited extent and depends on two factors according to our model: (1) the proportion of TB infected persons in the total population, which is comparatively high in South Africa with 0.52% (mean value of the 30 most severely infected countries is around 0.32%), and (2) the fatality rate of TB infected people, which is also high in South Africa at 21.2% (mean value of the 30 most severely infected countries is around 17%).

Calculations for the number of critical cases are based on recent studies on COVID-19, which may also differ from previous findings due to the age structure of African countries. A vulnerability factor depending on age^10^ may be applicable to our simulations once reliable estimates exist. The high number of young people in South Africa may reduce the number of ICUs accordingly. However, this mitigating age effect for developing countries may be thwarted by potential comorbidities^45^.

In this study we show that regional assessments with agent-based simulations for epidemiology questions are a valuable tool. Regarding vulnerable subpopulations, infection rate growth can be analysed in order to get a better estimation of potential effects of comorbidities. Accordingly, a population with a share of 0.52% has a high impact on critical cases with 8–14%, which should lead to a focus on these cases by decision makers to reduce the burden on the healthcare system.

Perhaps the most important goal of early identification of the impact of vulnerable subpopulation would be to employ adequate counter- and precautionary measures. A possible approach is a targeted isolation of high-risk patients to improve their health status before infection with COVID-19, in order to relieve the health system during the peak of the epidemic^46^. Our previous study^39^ but also further indication exists that the length of a measure may only delay or prolong COVID-19^47^. In addition countermeasures should consider human rights as their extent and their enforcement may inflict with human rights^47^.
